# Regulation of Immune System Cell Functions by Protein Kinase C

**DOI:** 10.3389/fimmu.2013.00384

**Published:** 2013-11-18

**Authors:** Noah Isakov, Amnon Altman

**Affiliations:** ^1^Faculty of Health Sciences, The Shraga Segal Department of Microbiology, Immunology and Genetics, Cancer Research Center, Ben Gurion University of the Negev, Beer Sheva, Israel; ^2^Division of Cell Biology, La Jolla Institute for Allergy and Immunology, La Jolla, CA, USA

**Keywords:** protein kinase C, T cell activation, signal transduction pathways, lymphocyte stimulation, cell growth regulation

Cellular responses to environmental cues are mediated through complex networks of signal transduction pathways. Among the molecules involved in these pathways, members of the protein kinase C (PKC) family stand out because of their ability to acutely and reversibly modulate effector protein functions and control the spatial distribution and dynamic properties of the signals. PKC enzymes also contribute to signaling networks that coordinate many aspects of immune cell function and, therefore, are important players in immune regulation.

Originally discovered in 1977, by Nishizuka and coworkers, PKC was initially identified as a cyclic nucleotide-independent protein kinase that is capable of phosphorylating histone and protamine following Ca^2+^-dependent limited proteolysis of its proenzyme precursor (1, 2). Subsequently, Nishizuka demonstrated that activation of PKC can occur in the absence of limited proteolysis by a membrane-associated factor and low Ca^2+^ concentration (3). He then characterized the membrane-associated factor as diacylglycerol, which is generated by receptor-stimulated hydrolysis of phosphoinositides, suggesting that this lipid functions as a second messenger (4). These findings and additional data established a new pathway of signal transduction and led to identification of biological roles for PKC in signaling pathways linked to a variety of surface receptors in many different cell types.

The PKC family of serine/threonine kinases consists of 10 distinct isoforms that are differentially expressed in a wide range of cell types and tissues. Despite having a certain degree of redundancy and overlapping substrate specificities, individual PKC isoforms also exhibit non-redundant functions. In addition, activity of distinct isoforms can be deployed in a spatially and temporally dependent manner since most isoforms possess a structurally and differentially activated unique regulatory domain. The activity of PKC can therefore be induced by a large variety of agonists and directed by multiple inputs, including second messengers and a variety of PKC-binding proteins.

This Research Topic focuses on recent developments relevant to the role of PKC in immune cell functions, and includes contributions by many of the leading experts in the field. The first paper by Pfeifhofer-Obermair and colleagues (5) reviews current information related to the physiological role and non-redundant functions of different PKC isoforms in T lymphocytes. The authors emphasize the positive contributions of PKCθ (6) and PKCα (7) to antigen-induced T cell activation and argue that inhibition T cell-mediated responses, including allograft rejection and autoimmunity, requires the inhibition of both PKC isoforms.

Black and Black (8) discuss mechanisms of regulation of T cell proliferation and cell cycle progression that are mediated by distinct PKC isoforms and emphasize their predominant role during the G0/G1 and G2 phases. Although PKC was found to modulate an array of cell cycle regulatory molecules, evidence points to Cdk kinase inhibitors and D-type cyclins as the key mediators of PKC-regulated cell cycle-specific effects. The authors indicate that most PKC isoforms play positive roles during cell cycle progression, except for PKCδ, which serves as a negative regulator.

Another important cellular function that involves PKC is the establishment and maintenance of cell polarity. This mechanism enables, among other things, the formation of a functional immunological synapse (IS) at the T cell-antigen presenting cell (APC) contact area, and the directional release of cytokines and cytolytic factors from cytotoxic T cells toward their specific target cells. The microtubule organizing complex (MTOC) plays an important role in directing this polarity, and the review by M. Huse covers the process through which PKC family members regulate the formation of the MTOC and its link to the T cell IS (9).

The PKCθ isoform, which is selectively enriched in T cells (10), is a key modulator of T cell receptor (TCR) signaling and an essential regulator of T cell activation and survival (11). In antigen-stimulated T cells, PKCθ selectively translocates to the center of the IS, where it mediates critical TCR and CD28 signals leading to the activation of NF-κB, AP-1 and NFAT transcription factors (12).

A review by Wang and coworkers (13) discusses a major mechanism that regulates PKCθ, and is also shared by other kinases. This mechanism involves a post-translational process whereby distinct serine, threonine, and tyrosine residues of PKCθ are autophosphorylated or transphosphorylated, thereby regulating its catalytic activity, as well as its localization within cells and the ability to interact with specific binding partners. The authors focus on the actual PKCθ phosphorylation sites and their potential role in determining PKCθ functions. A short comment on this topic is presented by Freeley and Long (14).

Another manuscript, by Isakov and Altman (15), focuses on the molecular mechanism that regulates the translocation of PKCθ to the center of the IS. This mechanism was discovered only recently by showing that a proline-rich region within the unique V3 (hinge) domain of PKCθ recruits the enzyme to the cytoplasmic tail of CD28 in a TCR activation-dependent manner (16). The PKCθ-CD28 interaction is mediated via an indirect mechanism involving the Lck protein tyrosine kinase as an intermediate. The authors review progress made in recent years in our understanding of the PKCθ-mediated signal delivery from the TCR/CD28 surface receptors.

Another facet of PKCθ relates to its nuclear function in T cells, where it was found to associate with chromatin in a cell activation-dependent manner. PKCθ interacts with selected nuclear proteins with which it forms active complexes that associate with proximal promoters of inducible T cell genes, including CD69, INF-γ, and TNF-α (17). The nuclear function of PKCθ and its potential involvement in specific transcriptional programs in T cells are discussed by Sutcliffe and colleagues (18).

In addition to the TCR, additional receptors, including OX40, a member of the tumor necrosis factor receptor (TNFR) superfamily, also utilize PKCθ for signal transmission, a topic that is presented in this issue by So and Croft (19). Following activation of T cell-expressed OX40 by its APC-expressed ligand, OX40L, activated OX40 delivers TCR-independent signals that promote optimal activation of NF-κB, using signaling intermediates that are distinct from those utilized by the TCR, e.g., TRAF proteins and RIP1.

PKCθ-deficient mice serve as an important tool for deciphering underlying mechanisms in cellular immune responses. While *in vitro* studies of PKCθ-deficient T cells demonstrated impaired activation responses, *in vivo* studies indicated that the requirement for PKCθ is not universal. Anel and colleagues (20) discuss the involvement of PKCθ in natural killer cell function and anti-tumor immunity, while the importance of PKCθ during the induction of graft-versus-host and graft-versus-leukemia responses, or in antiviral immunity is reviewed by Bronk and colleagues (21).

In another review, Sun (22) elaborates on the potential role of PKCθ in maintaining the normal balance between effector and regulatory T cells and the possibility of targeting PKCθ for intervention in T cell responses and prevention of selected autoimmune diseases and allograft rejection.

Both PKCη and PKCθ are members of the novel PKC subfamily that are highly expressed in T cells. However, in contrast to PKCθ, which is concentrated at the center of the IS in activated T cells, PKCη remains localized in a diffuse pattern over the entire IS, suggesting distinctive roles for these two isoforms in signal relay downstream of the TCR. Fu and Gascoigne (23) discuss the specific roles of PKCη and its functional redundancy with PKCθ in T cell biology.

Members of the atypical PKC subfamily (aPKC), including PKCζ and PKCι/λ, are also expressed in lymphocytes and play important roles in T and B cell differentiation, as well as in the regulation of T cell polarization and survival. A detailed description of the mechanism of action of aPKCs, in conjunction with their adapters, p62 and Par-6, in the PB-1-orchestrated signaling network that regulates lymphocyte behavior, is provide by Martin and Moscat (24).
